# Teachers’ working time as a risk factor for their mental health - findings from a cross-sectional study at German upper-level secondary schools

**DOI:** 10.1186/s12889-022-12680-5

**Published:** 2022-02-14

**Authors:** Steffi Kreuzfeld, Christoph Felsing, Reingard Seibt

**Affiliations:** Institute for Preventive Medicine of the Rostock University Medical Center, St.-Georg-Str. 108, D-18055 Rostock, Germany

**Keywords:** Full-time teachers, Long working hours, Workload, Methods of time recording, Upper-level secondary education, Inability to recover, Emotional exhaustion

## Abstract

**Background:**

The work of teachers has changed due to an increase in the range of tasks. However, there is a lack of current information on working hours, task distribution and the possible health effects.

**Methods:**

For the first time for Germany as a whole, a cross-sectional survey determined how long teachers at upper-level secondary schools work per week, what influences their working hours and how different recording methods affect the total working hours. To this end, 6,109 full-time teachers estimated their working hours based on twelve categories and then documented these daily over 4 weeks. Afterwards, the effects of long working hours on teachers' ability to recover and emotional exhaustion were analysed.

**Results:**

The article shows the large interindividual variance in the working hours of teachers and a significant influence of sex, age, and subject profile. Self-reported working hours varied substantially by method used to record working time with work time reported via daily diaries totaling 2 h per week more than hours recorded by a single estimation.

A substantial proportion of the teachers (36%) work longer per week than European guidelines allow (> 48 h); 15% work even more than 55 h per week. Teachers who work more than 45 h per week suffer more often from inability to recover (46%) and emotional exhaustion (32%) than teachers who work less than 40 h per week (26% and 22% respectively).

**Conclusions:**

Taking professional experience and teaching subjects into account could in future contribute to a fairer distribution of workload among teachers. This could protect individual teachers from long working hours, ensure sufficient recovery and also reduce the risk of emotional exhaustion. In order to identify teachers whose health is at risk at an early stage, voluntary preventive care offers would be considerably helpful.

**Supplementary Information:**

The online version contains supplementary material available at 10.1186/s12889-022-12680-5.

## Background

Researchers from different countries have long reported high workloads and time pressure as among the main factors associated with stress in the teaching profession [1–4]. Due to the complexity of the professional requirements, however, it is important to describe the workload more precisely in the form of suitable indicators, e.g. by the number of hours teachers need for a wide variety of tasks [5]. For decades, studies in Germany have provided indications that at least some teachers work excessively long weekly hours [6]. In a comparison of different types of schools (primary, lower-level secondary, upper-level secondary), it is noticeable that upper-level secondary school teachers work the longest of all teachers [7, 8]. However, there are no current statements on the total working hours and task structure of teachers at upper-level secondary schools across Germany. The only nationwide survey on working hours, which also included upper-level secondary school teachers and is considered representative took place in 1973 exclusively in the western part of Germany [9].

Long working hours can have a negative impact on well-being [10, 11] as well as physical and mental health [12–16]. Evidence suggests that risk of work-related stress and burnout increase with the number of working hours per week [17]. Nevertheless, it can be difficult for teachers to limit their working hours. Their own demands for good quality teaching and the expectations of pupils, parents, colleagues and the public force some of them to overcommit themselves, even though they realise that they are putting their health at risk. Many teachers work in the evenings and at weekends [18]. This not only reduces the time needed for recreation but also the possibility of detaching from work. However, this detachment from work is an essential prerequisite for recovery processes [19]. If teachers do not succeed in distancing themselves from the content of work, physiological activation which lasts longer than work can hinder recovery processes. As a result, the consequences of previous activities cannot be fully compensated for, so that the teacher's own performance reserves are chronically overtaxed [20]. In this respect, both long working hours and shortened regeneration times are significant for health [21, 22], especially with regard to exhaustion.

To reduce work-related stress and the risk of exhaustion, it is therefore necessary to record teachers’ working hours and range of tasks as precisely as possible and to recognise highly stressed colleagues at an early stage.

There is consensus that the working hours of teachers depend both on the working conditions (e.g. type of school, subjects taught) and on their individual skills and attitudes [7, 23–25]. However, data on the influence of sex and age on working hours are inconsistent [6, 26]. The factors mentioned have an effect in particular on the amount of time required for the preparation and follow-up of lessons as well as corrections.

In Germany and most European countries, the working hours of teachers are largely regulated by the number of mandatory teaching hours per week. Regulations in some countries specify attendance times at school as well as total weekly or annual working hours. Only in parts of Belgium and Liechtenstein is teachers’ working time exclusively defined by teaching hours; in the Netherlands and Denmark only the total annual working time is regulated by law [27, 28].

Nevertheless, in comparison to other occupational groups, it is difficult to determine the exact working hours of teachers, since teaching itself only makes up less than half of the total working time. The other part of the working time includes extra-curricular activities, some of which have to be performed at school (e.g. conferences, further training), but mostly at home (e.g. preparation and follow-up of lessons, correcting and grading student work). This proportion of working hours is to be organised independently and configured accordingly. In addition, working hours are subject to strong fluctuations over the week and year.

The proportion of time spent teaching averages 43% of total working time in OECD countries, but varies between 32% (Poland, Turkey) and 63% in Scotland [7]. Due to the increase in extra-curricular tasks, it has been observed that the proportion of time for actual teaching has decreased in the more recent studies [25, 29, 30].

In Germany, there have been repeated studies on the working hours of teachers in various types of school since the 1960s (overview: [6]). Since the findings from these were primarily written in German, they have hardly entered international literature. Two main methods of recording working hours have been used in Germany: on the one hand, the estimation of an average school week [23, 24, 31], and on the other hand, daily documentation based on pre-determined activity categories over a specified period of time [32–34].

The advantage of the estimation method over the diary method is that it is much less time-consuming. The total weekly working time is determined in the context of interviews or questionnaires either generally or differentiated, based on teacher-specific activity categories. A major disadvantage of the estimation method, however, is that current influences on working time (e.g. increased correction times) or sick days are not often considered.

Documentation of daily working time has been carried out using standardised protocols with defined activity categories, either as a paper version [9] as an electronic version [33] or as a browser-based recording system [32–34]. The number of specified job categories has ranged between two and 205 activities in the various studies.

For the acceptance and appropriateness of the working time protocols, it is crucial that the activity categories are understandable and unambiguous and that they reflect the entire spectrum of methodological-didactic, pedagogical, and administrative activities of individual teachers. At the same time, their number must be limited so that the documentation of the working hours remains clear and can be carried out within an acceptable amount of time. The definition of the activity categories therefore always represents a compromise between accuracy and the practicability of time recording.

Large, international studies by the OECD on the working time of teachers have used similar methods and found that the average documented working time tends to be higher when it is not only estimated with a single question, but also recorded in a more differentiated manner based on specified activities [35].

## Aim of the study

As part of a nationwide study at upper-level secondary schools in Germany the research therefore investigated how long full-time teachers currently work per week and which factors influence their working hours. For this purpose, two different recording methods (estimation and diary) were used and compared in terms of determining the total working time. Subsequently, this study examined whether longer weekly working hours are associated with teachers’ inability to recover or emotional exhaustion.

## Methods

### Recruitment of study participants

The cross-sectional study on the working hours of upper-level secondary school teachers (hereinafter: teachers) took place between January and April 2018 in all sixteen German federal states. In each federal state, a 4-week study period with average workload (no extraordinary activities due to exams, extensive corrections, class trips, etc.) was selected to ensure comparable conditions for working time recording nationwide. In the run-up to the study, posters and flyers were used to promote voluntary participation at all upper-level secondary schools. Immediately before the start of the study, all teachers in the school received an information letter on the study with information on data protection, implementation, and data evaluation as well as the conditions of participation and access to the study. Anonymity of data was ensured via transaction numbers (TANs) and an eight-digit personal code. This code was created according to predefined specifications and was only known to the participant. The data were collected via an online portal at the university.

The following groups of persons were excluded from participation: teachers on paternal leave, trainee teachers, teachers with long-term illnesses, teachers at vocational schools as well as temporary staff.

### Execution and data acquisition

Working time and activity distribution were determined in parallel using an online questionnaire (OQ) and an online working time protocol (in short: online protocol - OP). Both tools were developed by the authors for this study and used the same activity categories (see Additional files 1 and 2). In the operationalisation of the teacher-specific range of activities, the diverse tasks were transferred into practicable, suitably clear categories of activity. The determination of the categories was based on previous studies on teachers’ working hours [36]. The activity categories were restricted to twelve to develop a suitable variant for electronic recording on a mobile device.

First of all, the teachers used the OQ to estimate their working time for 1 week of average workload based on the defined activity categories (Table 1). At the same time, the OQ was used to document socio-demographic, job-specific as well as health-related data from the participants.

**Table 1:** Categories of teacher-specific activities

Category	Description
**Teaching**	
Lessons	Number of lessons
Substitute lessons	Number of lessons substituting for colleagues
**Teaching-related activities**	
Preparation / follow-up	Preparation (e.g. class tests, exams) and follow-up time of lessons
**Corrections / marks**	Correction and grading of pupils’ work (e.g. exams, homework)
Projects / excursions	Implementation of projects, excursions, class trips, pupil exchanges
**Non-teaching activities**	
Pupils / parents	Extracurricular work with pupils (e.g. pupil counselling, conversations regarding pupil education, communication) and co-operation with parents
Administration	Administrative tasks and organisational matters (e.g. certifications, planning of events, school trips or projects, orders)
Colleagues / teamwork	Teamwork and dialogue with colleagues, co-operation with colleagues
Inclusion	Tasks within the scope of pupils’ inclusion
Integration	Tasks within the scope of pupils’ integration
Supervision	Supervision times during breaks
All other tasks	All-day school activities, evaluations, safety, trainings, mentoring, commission memberships, staff council activities

The teachers then documented their working hours each day over a period of 4 weeks (28 days) using the activity categories in the OP. In addition, this included whether the teacher had been present at the school and had taught on each day of class. In the case of absence, the appropriate reason had to be selected (own illness / illness of relatives / regular class-free day / other personal or professional reasons). In the case of sick days, the teachers also indicated whether and to what extent schoolwork was carried out.

In order to simplify the documentation of working hours in everyday school life and to avoid distortion due to lapse of memory, the activities could first be recorded on paper (table of categories) and later transferred to the OP at any time.

### Mental health measurements

The health-related characteristics considered were inability to recover and emotional exhaustion.

*The inability to recover (IR)* is a subscale of the questionnaire for the analysis of stress-relevant coping requirements (German: FABA) [37] which describes the inability to cope with work-related stress to the detriment of one’s own recovery. IR is measured using six items on a four-point Likert scale (1 = does not apply at all to 4 = strongly applies). By summing the values (range: 6-24 points), interpretation into normal, noticeable and very noticeable is given [37]. The reliability of the subscale is rated as good, Cronbach’s alpha is given as 0.79 [38]. In the present study, a Cronbach’s alpha of 0.80 was determined for IR, which was considered acceptable and was at the limit of the good range [39].

*Emotional Exhaustion (EE)* was measured with the German translation of the Maslach Burnout Inventory-General Survey (MBI-GS) [40]. EE is one of three subscales of the MBI and is considered by many researchers to be the core component of burnout. It measures the frequency of occurrence of symptoms using five items on a seven-point Likert scale (0 = never up to 6 = daily). The mean value allows the classification into low, normal or high emotional exhaustion. The validity of the MBI-GS was proven by Schaufeli et al. [40]: Cronbach’s alpha was 0.87 for EE. For the burnout subscale EE of the study presented here, Cronbach’s alpha was 0.85, and thus, according to Blanz [39], it was in the range of good.

### Quality management of data collection

In October 2017, both recording methods were evaluated by eight teachers. The activity categories were then tested for comprehensibility, uniqueness, and freedom from errors in a preliminary study at four upper-level secondary schools in different federal states. A list of answers to frequently asked questions (FAQ) was then made available on the study website. In addition, there was the possibility of telephone and electronic queries to the team of investigators over the entire investigation period.

In both the OQ and in the OP, input aids and default settings prevented implausible time entries. Only study participants for whom both an OQ and an OP were available were included in the data analysis. Using the personal code, both documents could be merged for data evaluation. The completeness of the information in the OP was then checked. Participants who recorded their working hours on fewer than 21 out of 28 days were not included in the data analysis.

### Sample

A total of more than 20,000 upper-level secondary school teachers took part in the study. Of these, 18,791 filled out the complete OQ, so that complete data sets were available for all variables of these participants. This represents 11% of all secondary school teachers in Germany. In terms of sex and age, this sample was comparable with the total of all German upper-level secondary school teachers. Only the group of teachers over 60 was underrepresented (5% instead of 12%), and in terms of employment, part-time teachers had participated slightly more often than their share of all upper-level secondary school teachers (46% instead of 38%).

The OQ and OP codes matched and the quality requirements in the OP were satisfied by 14,338 participants. About 84% of these records (*n* = 12,014) were related to teachers who primarily give lessons. In contrast, 16% of the records referred to teachers who were employed as headmasters or deputy headmasters or who performed other administrative tasks and functions within the school to a considerable extent and therefore gave significantly fewer lessons. For comparison of the two recording methods reported here, a sample should be studied that was as homogeneous as possible. Therefore, only datasets from full-time teachers with a reduction of up to three hours (reduced teaching hours) were analysed (*n* = 6,109). The composition of the sample is shown in Table 2: women (56%) are slightly more often represented than men (44%).

**Table 2:** Characteristics of the sample

Variables	Number of teachers	%
**Full-time teachers**	6109	100
**Sex**		
- male	2680	43.9
- female	3429	56.1
**Age groups** [years]		
- 20 – 29	636	10.4
- 30 – 39	2442	40.0
- 40 – 49	1535	25.1
- 50 – 59	1177	19.3
- 60 – 67	319	5.2
**Employment**		
- civil servants^a^	5267	86.2
- other employees	842	13.8
**Subject profile**		
- languages	990	16.2
- social sciences	220	3.6
- natural sciences	1310	21.4
- languages and social sciences	1497	24.5
- languages and natural sciences	373	6.1
- social sciences and natural sciences	471	7.7
- art, music, sports	126	2.1
- subject combinations with art, music, sports	1122	18.4

Notes: %: frequencies in %

^a^In the state education system, many teachers gain the status of civil servants, which affords them certain rights and privileges

The average age is 41.3 ± 10.2 years and does not differ between the sexes. Half of the participants were between 20 and 39 years old. Science, languages and combinations of languages and social sciences are the most commonly taught subjects.

### Data processing

Prior to the statistical calculations, the entire dataset was checked for implausible information. The number of teaching hours and the reduction in hours (reduced teaching time) was checked in the OQ on the basis of information on age and the special tasks of the teacher. The range of time for the individual activity categories was examined in the OP for statistical outliers every 28 days. Extreme values were replaced by subject-specific mean values within the individual activity categories.

The times in the activity categories in the OQ and in the OP were indicated in minutes – with the exception of the teaching and substitute lessons (45-min units). To determine the total weekly working time, all information was converted into hours. In the OP, the average values over 4 weeks were calculated for all activity categories, provided there were no sick days (74% of the full-time data records). If there were any sick days documented, these weeks were not taken into account when calculating the average weekly working time. Instead, the average value was calculated from the remaining weeks without sick days. During the investigation period, 10% of the participating full-time teachers were sick for one day, another 14% were sick for two to five days and 2% were absent from work for six to ten days due to illness.

### Statistical analysis

The statistical analysis of the data recorded was carried out using the programme “Statistical Package for the Social Science” (SPSS, Version 25) for Windows. Differences between the working hours and activity categories of the OQ and OP were examined using general linear models with repeated measurements and univariate covariance analyses – controlled for sex, age groups and subject profile. Post-hoc tests (Bonferroni) were carried out for significant *p*-values. The Chi^2^ test was used to test the differences between categorical variables.

The significant level of all statistics was set at *p* < 0.05. Since even small differences produce significant results in large samples, effect sizes were determined. The partial eta squared (η^2^_partial_) was used to express the analysis of variance and Cohen’s d for interpretation the Chi^2^-test. Both effect sizes were interpreted according to the conventions of Cohen [41]. In the analyses of variance and the Chi^2^ tests, small effect sizes from η^2^_partial_ = 0.01 or d = 0.20 are considered statistically significant effects.

To highlight the relationship between weekly working hours and the health-related characteristics (inability to recover and emotional exhaustion), teachers were divided into two (extreme) groups: those working less than 40 h per week (*n* = 1,796, 29%) and those working more than 45 h per week (*n* = 2,983, 49%). This division is based on the one hand on the labor law requirement for full-time employment (40 h/week) and on the other hand on the fact that full-time teachers in Germany work an average of 45 h per week [42]. The third group, in which teachers work between 40-45 h per week (*n* = 1,330, 22%), was consciously excluded from the analyses.

Differences between the two extreme groups were examined for the characteristics of working hours and health with the help of univariate variance analyses and Chi^2^ tests, and for correlations between working hours and health-related characteristics with the Pearson product-moment correlation. Correlation coefficients were interpreted according to Bühl [43], where r ±≤0.10 is considered independent of each other.

## Results

### Influence of the method and the covariables on weekly working time

The amount of weekly working time (WWT) differs significantly between the two methods of data recording (*p* = 0.001; small effect) (Table 3). With the OQ, the sample examined estimates the average WWT at 42.8 h. However, if the WWT is determined by OP, the WWT is 45.2 h. This results in a significant difference of 2.4 h compared to estimated working time (*p* < 0.001).

**Table 3:** Main effects of the covariates on the weekly working time (WWT) of full-time teachers (*n* = 6,109)

Variables	WWT Online Questionnaire M ± SD [h]	WWT Online Protocol M ± SD [h]	F-value	*p*-value	η^2^_partial_
	42.8 ± 8.1	45.2 ± 8.7	51.50	0.001	0.017
Covariates					
Sex			40.39	<0.001	0.007
Age group			42.15	<0.001	0.007
Subject profile			7.96	0.005	0.001
Interaction effects					
Method * sex			8.51	0.001	0.003
Method * age group			3.62	0.027	0.001
Method * subject profile			4.04	0.018	0.001

Notes: *M ± SD* mean ± standard deviation. Online Protocol: mean of illness-free weeks. General linear model with measurement repetition; internal subject design: method; design: constant term + sex + age group + subject profile; test variable: F-value; df = 2; error def = 6,104; *p*-value: significance (two-sided); η^2^_partial_: partial eta square (effect size): 0.010 - 0.060 = small effect, <0.010 = none effect [41]

The results for the working time are also influenced by a significant sex and age effect and by the subject profile; these effects occur regardless of the recording method (Tables 3 and 4).

**Table 4:** Estimated versus documented weekly working time of full-time teachers (*n* = 6,109)

Covariates	Online Questionnaire	Online Protocol
	M ± SD [h]	M ± SD [h]
**Sex** (number of teachers)		
- male (2680)	41.9 ± 7.8	44.2 ± 8.6
- female (3429)	43.5 ± 8.2	45.7 ± 8.7
F-value	61.37	18.24
*p*-value (η^2^_partial_-value)	<0.001 (0.010)	<0.001 (0.012)
**Age groups** [years] (number of teachers)		
- 20 – 29 (639)	44.7 ± 7.9	47.0 ± 8.8
- 30 – 39 (2442)	42.9 ± 8.3	45.2 ± 8.8]
- 40 – 49 (1535)	41.9 ± 7.6	44.6 ± 8.6
- 50 – 59 (1177)	43.1 ± 8.1	44.8 ± 8.5
- 60 – 67 (316)	41.0 ± 8.3	42.2 ± 8.3
F-value	18.75	42.06
*p*-value (η^2^_partial_-value)	<0.001 (0.012)	<0.001 (0.007)
**Subject profile** (number of teachers)		
- languages (990)	44.8 ± 8.5	46.5 ± 9.2
- social sciences (220)	43.7 ± 9.3	44.6 ± 9.3
- natural sciences (1310)	41.9 ± 7.7	44.7 ± 8.3
- languages and social sciences (1497)	43.2 ± 8.3	45.1 ± 8.9
- languages and natural sciences (373)	43.1 ± 7.8	45.2 ± 8.4
- social sciences and natural sciences (471)	42.5 ± 7.4	44.7 ± 8.2
- art, music, sports (126)	39.2 ± 6.7	42.0 ± 7.6
- subject combinations with art, music, sports (1122)	41.6 ± 7.7	42.2 ± 8.6
F-value	19.96	7.13
*p*-value (η^2^_partial_-value)	<0.001 (0.022)	<0.001 (0.008)

Notes: *M ± SD* mean ± standard deviation. Univariate variance analyses; test variable: F-value; df = 2; error def = 6,104; post-hoc test: Bonferroni; *p*-value: significance (two-sided); η^2^_partial_: partial eta square (effect size): 0.010 - 0.060 = small effect, < 0.010 = none effect [41]

On average, women work significantly longer per week than men (*p* < 0.001; small effect). Regarding the age effect, it should be noted that younger colleagues (20 - 29 years) work the longest (OP: 47.0 h/week). Older colleagues (60 - 67 years) on the other hand work the least amount of time (OP: 42.2 h/week). This effect could be demonstrated in both recording methods.

In addition, WWT are significantly influenced by the subject profile (*p* < 0.001; small effect). Teachers who teach one or more languages, regardless of the method, document the longest WWT (OP: 46.5 h/week), followed by the subject profile from languages and natural or social sciences (OP: >45 h/week) (Table 4).

For the average WWT (Table 4), high deviations are noticeable up and down in the form of standard deviations. The differences in WWT between teachers are therefore up to 18.6 h per week. On the basis of the recorded data (OP), 49 % of the teachers (*n* = 2,983) worked more than 45 h per week during the observation period. About 36 % of the teachers (*n* = 2,199) exceeded the European working time limits of 48 h weekly, 11 % (*n* = 672) worked more than 55 and 4 % (*n* = 244) even more than 60 h weekly.

### Correlation between working time and health-related characteristics

**Fig. 1:**
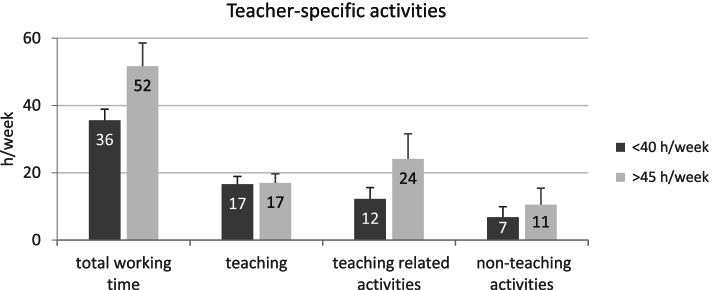
Comparison of the mean number of hours per week for teachers who work less than 40 or more than 45 h per week

In order to comprehend what causes the considerable differences in WWT between individual teachers, the volume of teacher-specific activities of the group of teachers who work less than 40 h per week (29%) was compared to the group of teachers who work more than 45 h per week (49%) (Fig. 1). The difference in mean weekly working hours between the two groups is remarkable and comes to about 16 h, which corresponds to two normal working days. It is evident that it is mainly the extent of teaching-related activities that differs between the working time groups (η^2^_partial_ ≥ 0.14, large effects). This category includes all activities of preparation and the follow-up of lessons and projects as well as correction work.

In the correlation analysis, the categories teaching, teaching-related and non-teaching activities show only very low correlations with each other (r = 0.04 to -0.15). Accordingly, the amount of time invested in teaching-related and non-teaching activities does not depend on the number of teaching hours.

Table 5 compares the manifestations of the health-related characteristics for the two working time groups. The group of teachers working less than 40 h per week differs from the group with weekly working hours of more than 45 h by a significantly increased inability to recover (d = 0.41, medium effect) and a significantly higher emotional exhaustion (d = 0.23, small effect). When working more than 45 h per week, almost half of the teachers (46%) show noticeable recovery values and one third of the teachers (32%) show high exhaustion values, whereas in the group with a WWT of less than 40 h, this only applies to one quarter (26% and 22% respectively) of the teachers. This means that with a WWT of more than 45 h, only every second teacher succeeds in recovering sufficiently from the workload.

**Table 5:** Comparison of inability to recover and emotional exhaustion depending on weekly working hours

Variables	Weekly working hours of teachers [h]						Significance	
	Total (*n* = 4779)		<40 (*n* = 1796)		>45 (*n* = 2983)		χ^2^-value	*p*-value (effect size d)
	n	%	n	%	n	%		
**Inability to recover** (IR) [6 – 24 pts]								
*- normal* (6 - 18 pts)	2935	61.4	1328	73.9	1607	53.9	196.28	<0.001 (0.414)
*- noticeable* (19 - 20 pts)	895	18.7	252	14.0	643	21.6		
*- very noticeable* (≥21- 24 pts)	949	19.9	216	12.0	733	24.5		
**Emotional exhaustion** (EE) [0 – 6 pts]								
*- low* (≤2.0 pts)	2226	46.6	982	54.7	1244	41.7	83.99	<0.001 (0.268)
*- normal* (>2.0 – <3.2 pts)	1186	24.8	413	23.0	773	25.9		
*- high* (≥3.2 – 6.0 pts)	1367	28.6	401	22.3	966	32.4		

Notes: *n* number of teachers, *pts* points; %: frequency in %. Chi-square test (Pearson); test size: *χ*^2^-value; *p*-value: significance (two-sided); effect size d: 0.2 - 0.4 = small effect [41]

However, the correlation analyses only partially support these statements, because only 8% of the health characteristics tested here can be explained by the different amount of working time. The clearest correlation is between weekly working hours and the ability to recover (r = 0.29), with a trend of increased weekly working hours being associated with increased inability to recover. For emotional exhaustion, this correlation is very low (r = 0.18). Ability to recover and emotional exhaustion correlate moderately (r = 0.59) with a variance explanation of 35 %.

## Discussion

### Long working hours and effects on mental health

The results of the study show that several teachers at German upper-level secondary schools work long hours per week even in phases of average workload. More than a third of the teachers examined exceed the statutory maximum work limit of 48 h per week. Over 15% of teachers document weekly working hours above 55 h, a limit that is associated with an increase in the risk of cardiovascular diseases and depression [15, 44].

The long weekly working hours are mainly the result of the individual workload for tasks that have to be done in addition to the 17 compulsory teaching hours. These tasks are often conducted by teachers at home in the evenings and at weekends. This can make it difficult to mentally detach from work. However, this detachment is an important precondition for successful recovery, which is considered a health resource [19, 45, 46].

Compared to other occupational groups, teachers seem to have less success in mentally detaching themselves from work. Based on a representative survey of German employees, Varol et al. [47] showed that teachers reported difficulties in mentally detaching from work twice as often as other professions (42 % vs. 21 %). The main reasons for this were emotional demands as well as time and performance pressures. Overall, teachers were the second most frequently affected (23%) by recovery problems after managers [48].

The overall weak correlations between working hours and recovery ability or emotional exhaustion in this study suggest that teachers manage their job demands differently and that working hours are less significant than expected. Whether a high number of working hours ultimately becomes a health risk probably depends more on the individual's way of coping these demands. Inability to recover is seen as an individual coping pattern in dealing with job demands and is associated with high work commitment and the acceptance of limited recovery [49]. Teachers with this coping pattern are likely to be more at risk of overestimating their own reserves and suffering from exhaustion.

The data show that teachers who work more than 45 h per week are significantly more frequently affected by signs of inability to recover and emotional exhaustion compared to those who work less than 40 h per week. The results presented offer additional evidence that long working hours inhibit recovery processes and increase the risk of emotional exhaustion [17, 50, 51].

### Influence of the recording method

The results suggest that the method used to record working time is important. The teachers estimated their average weekly working time as significantly shorter (42.8 h) than they documented in a subsequent four-week period by daily log (45.2 h).

It seems to be difficult to estimate the WWT of an average school week. This is not surprising when analysing the documented working hours. No school week resembles any other one. Even the number of lessons varies considerably from week to week, despite the fixed number of compulsory lessons. In addition to the absence of colleagues due to illness, excursions as well as further trainings and extra-curricular activities, all lead to unscheduled changes in the weekly distribution of hours. Such changes cannot be taken into account when estimating an average WWT. In this respect, a differentiated daily documentation of individual work activities appears to be more reliable in correctly recording WWT. This view is also held by other authors [8, 52, 53].

Juster and Stafford [54] postulate that the working hours of an average week can only be validly estimated if daily work patterns have regular schedules, which is rarely the case for teachers. But it is also apparently difficult for other occupational groups to estimate the hours of an average working week. Compared to diary documentation, almost all employees from the most varied of occupational groups overestimated their working hours by 3 h or more per week [55, 56]. Contrarily, the fact that teachers tend to underestimate their working hours may be due to their significantly higher weekly working hours during school periods than most of the general population.

Current studies from Germany [34] and Switzerland [29] used selected samples with considerable effort to implement the day-to-day documentation of working hours over an entire educational year (total survey). This form of working time documentation is the gold standard for determining teachers’ working hours [54] and is necessary to map fluctuations in the workload within a year as well as to record weekend and holiday working time. However, the personnel and financial effort required for such a total survey is immense.

Despite the diversity of the recording methods used over six decades, Hardwig and Mußmann [6] found surprisingly high agreement of the central findings on working time after a meta-analysis of 20 working time studies for Germany. They concluded “... that well-founded estimation procedures ... can certainly lead to sufficiently precise results if full surveys provide orientation at greater intervals” [ibid. p. 94].

### Influence of sex, age and subject profile on working time

Although the study dealt with a clearly defined sample of full-time, upper-level secondary school teachers, a large variance in the documented WWT (SD = 8 - 9 h/week) was observed regardless of the method used. This finding once again confirms the phenomenon known for some time that individual working hours in the teaching profession are very variable and depend on many factors. As observed, dispersion in the WWT can reach a considerably higher level, even within a school type. For example, Jerrim and Sims [35] reported differences in working hours of up to 37 h per week for English full-time, lower-level secondary school teachers. For German upper-level secondary school teachers, Mummert [57] demonstrated that – over a whole year – there are clearly more than 600 h of work between those who work a lot or very little [ibid., p. 40].

In the study presented here, sex, age, and subject profile emerged as relevant influencing factors for the working hours of teachers. Female teachers work significantly longer than male teachers. In the OP female teachers documented an average of 1.5 h more working time per week than their male colleagues. This is a remarkable result since previous studies in Germany have reached contradictory conclusions as to whether sex is an influencing factor and, if so, in which direction [6]. The main reason for this contradiction is seen as an overlap of the sex effect with influencing factors such as part-time work, school type and additional functions. To control such confounders, analyses only included data from full-time teachers at upper secondary schools with up to a three-lesson reduction for additional functions. Also, the teaching obligation for male and female teachers was comparatively high in this study.

The age effect also occurs regardless of the method of recording. It is most evident in the difference of 5 h per week on average between younger teachers and those just starting (20 - 29 years, WWT = 47 h) and the oldest colleagues (60 - 67 years, WWT = 42 h). It should be noted that 60% of teachers in the highest age group are allocated one to three fewer lessons per week (age-related reduction) and thus partly save time in the preparation and follow-up of lessons. The number of lessons per week therefore for those aged 60 and over differs by at least 2 h on average from all other age groups examined. It can be assumed that young professionals need more time than their more experienced colleagues in preparing lessons, and this time is reflected in the WWT. The observation is in turn supported by findings from the TALIS study: early-career teachers with fewer than five years of professional experience work 2.6 h per week more than those with 6 to 10 years of professional experience and 4.7 h per week more that those who have been teaching for more than 10 years [26]. This finding is viewed critically because the risk of a career exit is particularly high in the first five years [58].

It is noteworthy that teachers in the age range of 30 and 59 years, on the other hand, almost uniformly document their average WWT in the OP at around 45 h, although different proportions of them (54 - 64%) receive a reduced allocation of hours. For English teachers in secondary schools, Micklewright et al. [59] also come to the conclusion that WWT does not differ in the middle age groups and is between 45 and 47 h on average. Knight Wegenstein [9], however, indicates that a shortening of lesson preparation due to experience was not observed especially among teachers at upper-level secondary schools. Gehrmann [31] describes a working time plateau at around 40 years of age and a drop in WWT after the age of 60 for all school types but considers the influence of age to be low overall [ibid. p. 305]. However, he puts forward that thesis that teachers, regardless of age, limit their WWT to about 44 h in a self-regulatory process, at the expense of teaching quality [ibid., p. 312 ff] and that if the obligation to teach increases, time for lesson-related activities is reduced.

In relation to the influence of the subject profile, the here presented study shows the highest WWT for teachers who only teach a language profile, followed by those with a social or scientific profile. By contrast, teachers who teach an artistic profile or sport document significantly shorter working hours. The influence of the subject on the total working time is mainly attributed to the subject-dependent effort for the preparation of lessons and the amount of correction [25, 56]. However, the combination of first and second subject is decisive for the total working time. The results of the study fit into a largely consistent knowledge base of German and international studies on teaching hours based on subjects taught [6, 25].

### Strength and limitations

To the best of our knowledge, this study is the first to present data on the working hours of full-time, upper-level secondary school teachers for the whole of Germany, taking into account significant influencing factors and potential health risks. The sample is characterised by a homogeneous collective compared to other teacher studies. A mix of teachers with functionaries (e.g. staff councils) and principals has been consistently avoided. The results confirm on the one hand the known influence of teaching subjects on WWT and on the other hand add new knowledge on the influence of sex and age on working hours. In addition, it makes clear that total working time is influenced by the recording method.

The results presented here should be interpreted in the light of the limitations of this study. Since participation in the study was voluntary, selection effects may have had an impact. For example, the proportion of male school teachers in the study was 8% lower than the proportion of all male, full-time, upper-level secondary school teachers in Germany. The documentation of working times represented an additional time burden for the teachers, which could have acted as an obstacle in the recruitment of participants. Therefore, it is possible that teachers with heavy professional workloads and private burdens did not take part in the study due to lack of time.

From a methodological perspective, it should be noted that the working time of the teachers was only recorded over a period of 4 weeks and thus only presents an excerpt from the school year. Third subjects taught were not taken into account when creating the subject profiles. It was assumed that this was only a minor subject with a low teaching obligation.

Personality traits related to work behaviour, such as self-efficacy, conscientiousness and unhealthy perfectionism, were not studied in this paper. This leaves open the question of the extent to which they can have an influence on working hours. Finally, the data collection was conducted as a cross-sectional study, which prevents the findings of causal relationships between independent variables.

## Conclusions

The present results underline the need to consider essential factors influencing the working hours of teachers, both in research and in educational policy decisions. There can be considerable differences in the weekly working hours of teachers depending on professional experience and subject profile. However, excessively long working hours may harbour health risks and lead to a loss of quality in teaching. There is also a risk of early career abandonment for young professionals.

Therefore, working conditions should be arranged in such a way that the volume of work tasks can be managed by any teacher in a reasonable amount of time. A fair distribution of work tasks and opportunities to recover from work regularly and sufficiently are essential health resources for teachers. In addition, teachers should acquire competencies in professional self-regulation from the very beginning of their education. This is an important precondition for successfully coping with the subsequent requirements of the profession. Due to its high demands on flexibility and autonomy, the teaching profession involves the risk of being overburdened. From a preventive medicine perspective, the recording of working time can be a form of individual control that supports teachers in not extending their working hours to an extreme number in order to be able to recover sufficiently.

## Supplementary Information

**Additional file 1.** Online Questionnaire (acquisition of socio-demographic and job-related data).

**Additional file 2.** Online Protocol (recording of daily working time).

## Data Availability

The dataset supporting the conclusions of this article is not included within the article on the basis of agreed data protection commitments to the participants.

## Abbrevations

OQ: Online Questionnaire
OP: Online Protocol
IR: Inability to Recover
EE: Emotional Exhaustion
MBI-GS: Maslach Burnout Inventory - General Survey
