# Supplementary material for: Teachers’ working time as a risk factor for their mental health - findings from a cross-sectional study at German upper-level secondary schools
Source: BMC Public Health. 2022 Feb 14;22:307. doi: 10.1186/s12889-022-12680-5 (PMC8845294; doi:10.1186/s12889-022-12680-5)
Supplement: Supplementary file 1 — Additional file 1. Online Questionnaire (acquisition of socio-demographic and job-related data). [file 12889_2022_12680_MOESM1_ESM.pdf]

Online questionnaire

Dear teachers, dear colleagues,

This questionnaire contains questions about your current **personal** and **work-related situation**. The questionnaire completes the working time protocol and examines the extent of the working load at German upper-level secondary schools.

First of all, please tick in the following table in which **federal state** you teach!

1	Baden-Württemberg	5	Bremen	9	Niedersachsen	13	Sachsen
2	Bayern	6	Hamburg	10	Nordrhein-Westfalen	14	Sachsen-Anhalt
3	Berlin	7	Hessen	11	Rheinland-Pfalz	15	Schleswig-Holstein
4	Brandenburg	8	Mecklenburg-Vorpommern	12	Saarland	16	Thüringen

General information about your professional activity

Some questions may not apply exactly to your situation, as not all country-specific characteristics can be covered in one questionnaire. Nevertheless, please **always give an answer**, namely **the one** which, in case of doubt, **applies most closely to you**.

Please note that your answers sometimes refer to **teaching hours** (á 45 minutes) and sometimes to **time hours** (á 60 minutes)**!**

1.1 Have you **studied grammar school teaching** or do you have a **teaching qualification** (according to the law of the former GDR) **up to the university entrance qualification** (Abitur)?

☐$_0$ no ☐$_1$ yes

Have you studied **another type of teaching**?

☐$_0$ no ☐$_1$ yes

If **both questions** are answered **negative:**

What did your transverse entry look like?

☐$_1$ Trainee teacher

☐$_2$ Further training

☐$_3$ Other entry: what? Please specify! _______

If **a question** is answered with **yes:**

For which **school subjects** are you **trained**?

Subject 1: ___________________________

Subject 2: ___________________________

Subject 3**:** ___________________________

1.2 For how many **years ...**

- have you been **working as** a **teacher** (not including your traineeship)? _____ years
- have you been **working at** your **current school**? _____ years

---

1.3 In which **type of employment** are you employed?

☐$_1$ Civil servants ☐$_2$ Other employees

1.4 Is this **employment** a ...

☐$_1$ Permanent employment? ☐$_2$ Temporary employment?

1.5 What is the **number of compulsory hours** for you in a **full-time** position?
(without credit / reduction hours) _____ lessons

1.6 **Please imagine a "normal" school week in the school year.** What is your **weekly number** of ...

- Lessons: _____ lessons (á 45 minutes)
- Substitute lessons: _____ lessons (á 45 minutes)
- Free lessons (free periods between lessons) _____ lessons (á 45 minutes)

- **Preparation** (e.g. class tests, exams) and **follow-up** time of lessons (without corrections) _____ hours
- **Corrections** (e.g. class tests) and **grading of pupils' works** (e.g. exams, tests, homework, subject-specific works) _____ hours
- **Time** required to carry out **projects**, **excursions**, **class trips, pupil exchanges** _____ hours

- **Extracurricular work** with **pupils** (e.g. pupil counselling, conversations regarding pupil education, communication) and **co-operation** with **parents** (e.g. parents' evenings, class activities, presentation days) _____ hours
- **Administrative tasks** and **organisational matters** (e.g. certifications, planning of events, class trips or projects, orders, protocols, certificate conferences, archiving exams) _____ hours
- **Teamwork** and **dialogue** with **colleagues**, **co- operation** with **colleagues** (e.g. expert conferences, expert discussions, arrangements) _____ hours
- **Tasks** within the scope of pupils' **inclusion** (e.g. preparation and follow-up time of lessons, specific advanced trainings, internal differentiation) _____ hours
- **Tasks** within the scope of pupils' **integration** (e.g. preparation and follow-up time of lessons, specific advanced trainings, internal differentiation) _____ hours
- **Supervision** times during breaks _____ hours
- **All other tasks** (e. g. all-day school activities, evaluations, safety officer, maintenance of technology, training courses, mentoring, commission membership, staff council activities, care of a subject collection, use of advisory services and public authorities, work as subject chairperson, etc.) _____ hours

1.7 Do you work **part-time / partial retirement**? ☐0 no ☐1 yes

If you work **part-time** / **partial retirement**:

How many **years** have you been working **part-time / partial retirement**? _____ years

What is the **main reason** for this?

☐1 High workload
☐2 Health reasons
☐3 High importance of my free time
☐4 High private burdens (e.g. childcare, care of a relative)
☐5 Other reason: what? Please specify!
____

Specific information about your teaching activities

2.1 **Which of the following tasks are you responsible for in addition to teaching?**
(multiple answers possible)

☐0 **No** additional tasks

☐1 **Headmaster/ Deputy Headmaster**

☐2 **Coordinator** (member of the school management or **of the extended school management** (e.g. head of the upper school)

☐3 **Functional position beyond the scope of the school** (e.g. expert advisor, expert leader in training)

☐4 **Additional tasks with or without a functional position** (e.g. safety officer, equipment maintenance, subject supervisor, collection manager)

☐5 **Head of class, tutor**

☐6 **Other tasks** (e.g. school organisation, work in school committees)
What? Please specify! ________________________

2.2 How much **time per week** do **these tasks** (question 2.1) take in a **'normal' school week in the school year?** _____ hours

2.3 **How many credit / reduction hours do you receive for these tasks** (question 2.1)?
_____ lessons (no reduction hours = 0 lessons)

2.4 Do you receive **credit / reduction hours** (e.g. age reduction, certain subjects, further training) **independently of these tasks** (question 2.1)? ☐0 no ☐1 yes

If **yes,** how **many** and for **what**:

Number of lessons	Credit / reduction hours for …
e.g. 2	*e.g. age reasons*

2.5 **Please provide the following information about the classes you teach.** How **many** … **Number**

- **classes** do you teach in a **normal school week**? ______
- **pupils** are there in a **class on average**? ______
- **pupils** do you teach with a **migration background** without sufficient knowledge of German? ______
- **pupils** do you teach in the context of **inclusion**? ______

2.6 How many **additional hours** do you need per week for **inclusion tasks** (e.g. preparation and follow-up, differentiation within a class, tutoring)? _____ hours

How many **additional hours** do you need per week for **integration tasks** (e.g. preparation and follow-up, differentiation within a class, tutoring)? _____ hours

---

2.7 What **subjects** do you **teach?**

Subject 1: ________________________ Subject 3: ________________________

Subject 2: ________________________ Subject 4: ________________________

2.8 **Have you been employed in the last two years in a subject, for which you are not trained?**

☐$_0$ no ☐$_1$ yes **If yes:** At your own request? ☐$_0$ no ☐$_1$ yes

---

2.9 According to your **timetable**, how much **time per week** are **"long" breaks** (= longer than 10 minutes)**?** (without free, intermediate and super-visory hours) _____ minutes

How much **time per week** do you use **for recreation**? _____ minutes

How many **free, intermediate hours** do you have in your timetable **per week**? _____ lessons

How many **free, intermediate hours** do you use **for recreation per week**? _____ lessons

Do you need **more recreation time** in your school day? ☐$_0$ no ☐$_1$ yes

---

2.10 **Are you currently participating in a regular, job-specific further education?** ☐$_0$ no ☐$_1$ yes

If yes: How many **hours per month** do you invest in it? _____ hours

---

General information about yourself and your free time

3.1 **Sex:** ☐$_1$ male ☐$_2$ female **Age:** ____ years

3.2 **Marital status:** ☐$_1$ single ☐$_2$ married ☐$_3$ widowed.. ☐$_4$ divorced

Currently living in a stable partnership: ☐$_0$ no ☐$_1$ yes

3.3 **Do you have children?** ☐$_0$ no ☐$_1$ yes

If **yes:** How many children are **currently** living in your household? _____ children

---

3.4 **Are you currently caring for a relative?** ☐$_0$ no ☐$_1$ yes

If **yes:** How much **time** does this take **per week**? _____ hours

---

3.5 Do you **regularly** engage in **voluntary activities** (e. g. in clubs, associations, churches, parties or other organisations)? ☐$_0$ no ☐$_1$ yes

If yes: How much **time** does this take **per week**? _____ hours

---
