# Supplementary material for: Teachers’ working time as a risk factor for their mental health - findings from a cross-sectional study at German upper-level secondary schools
Source: BMC Public Health. 2022 Feb 14;22:307. doi: 10.1186/s12889-022-12680-5 (PMC8845294; doi:10.1186/s12889-022-12680-5)
Supplement: Supplementary file 2 — Additional file 2. Online Protocol (recording of daily working time). [file 12889_2022_12680_MOESM2_ESM.pdf]

Working time protocol

Instructions for filling in the working time protocol

- **Please enter compulsory lessons and substitute lessons as the number of lessons!**

  Please first indicate the **duration of a lesson** at your school.

  ☐ 45 minutes
  ☐ 60 minutes
  ☐ 75 minutes
  ☐ 80 minutes
  ☐ other duration of a lesson   Please specify: ______ minutes

- Please enter **extracurricular work tasks** in **minutes**.
- If **certain work tasks** can be assigned to **more than one activity category**, please **choose one** and write down the **time only once**! If **certain work tasks do not occur** on a day, these **boxes remain empty**!

Category	Description
**Lessons**	Number of **lessons**
**Substitute lessons**	Number of **lessons substituting** for colleagues
**Preparation / follow-up** (minutes)	**Preparation** (e.g. class tests, exams) and **follow-up time of lessons** (without corrections)
**Corrections / marks** (minutes)	**Corrections** and **grading of pupils' works** (e.g. exams, tests, homework, subject-specific works)
**Projects / excursions** (minutes)	**Time** required to carry out **projects, excursions, class trips, pupil exchanges**, etc.
**Pupils / parents** (minutes)	**Extracurricular work** with **pupils** (e.g. pupil counsellings, conversations regarding pupil educa-tion, communications) and **co-operation** with **parents** (e.g. parents' evenings, class activities, presentation days)
**Administration / organisation** (minutes)	**Administrative tasks** and **organisational matters** (e.g. certifications, planning of events, class trips or projects, orders, protocols, certificate conferences, archiving exams)
**Colleagues / teamwork** (minutes)	**Teamwork** and **dialogue** with **colleagues, co-operation** with **colleagues** (e.g. expert confer-ences, expert discussions, arrangements)
**Inclusion** (minutes)	**Tasks** within the scope of pupils' **inclusion** (e.g. preparation and follow-up time of lessons, specific advanced trainings, internal differentiation)
**Integration** (minutes)	**Tasks** within the scope of pupils' **integration** (e.g. preparation and follow-up time of lessons, specific advanced trainings, internal differentiation)
**Supervision** (minutes)	**Supervision** times during breaks
**All other tasks** (minutes)	**All other tasks** (e.g. all-day school activities, evaluations, safety officer, maintenance of technol-ogy, training courses, mentoring, commission membership, staff council activities, care of a sub-ject collection, use of advisory services and public authorities, work as subject chairperson, etc.)

If you were absent from school on any day, please write the applicable reason in the **ABSENCE** box:

- **1 = illness**
- **2 = care** (illness of children or relatives)
- **3 = day off from lessons / part time**
- **4 = other reason** (for personal or official reasons, e.g. training courses, class trip, pupil exchange)

In **case of attendance**, the **box** remains **empty**!

Work tasks	Weekdays						
**Week 1**	Monday	Tuesday	Wednesday	Thursday	Friday	Saturday	Sunday
**ABSENCE**							
Lessons							
Substitute lessons							
Preparation / follow-up (minutes)							
Corrections / marks (minutes)							
Projects / excursions (minutes)							
Pupils / parents (minutes)							
Administration / organisation (minutes)							
Colleagues / teamwork (minutes)							
Inclusion (minutes)							
Integration (minutes)							
Supervision (minutes)							
All other tasks (minutes)							

Work tasks	Weekdays						
**Week 2**	Monday	Tuesday	Wednesday	Thursday	Friday	Saturday	Sunday
**ABSENCE**							
Lessons							
Substitute lessons							
Preparation / follow-up (minutes)							
Corrections / marks (minutes)							
Projects / excursions (minutes)							
Pupils / parents (minutes)							
Administration / organisation (minutes)							
Colleagues / teamwork (minutes)							
Inclusion (minutes)							
Integration (minutes)							
Supervision (minutes)							
All other tasks (minutes)							

If you were absent from school on any day, please write the applicable reason in the **ABSENCE** box:

- **1 = illness**
- **2 = care** (illness of children or relatives)
- **3 = day off from lessons / part time**
- **4 = other reason** (for personal or official reasons, e.g. training courses, class trip, pupil exchange)

In **case of attendance**, the **box** remains **empty**!

Work tasks	Weekdays						
**Week 3**	Monday	Tuesday	Wednesday	Thursday	Friday	Saturday	Sunday
**ABSENCE**							
Lessons							
Substitute lessons							
Preparation / follow-up (minutes)							
Corrections / marks (minutes)							
Projects / excursions (minutes)							
Pupils / parents (minutes)							
Administration / organisation (minutes)							
Colleagues / teamwork (minutes)							
Inclusion (minutes)							
Integration (minutes)							
Supervision (minutes)							
All other tasks (minutes)							

Work tasks	Weekdays						
**Week 4**	Monday	Tuesday	Wednesday	Thursday	Friday	Saturday	Sunday
**ABSENCE**							
Substitute lessons							
Preparation / follow-up (minutes)							
Corrections / marks (minutes)							
Projects / excursions (minutes)							
Pupils / parents (minutes)							
Administration / organisation (minutes)							
Colleagues / teamwork (minutes)							
Inclusion (minutes)							
Integration (minutes)							
Supervision (minutes)							
All other tasks (minutes)							
